# Haemophagocytic Lymphohistiocytosis Following Ipilimumab and Nivolumab in Malignant Pleural Mesothelioma: A Case Report

**DOI:** 10.7759/cureus.112768

**Published:** 2026-07-16

**Authors:** Cho May Than, Maung Moe, Ye Win, Kyaw Maung

**Affiliations:** 1 Acute Medicine, United Lincolnshire Hospitals NHS Trust, Boston, GBR; 2 Oncology, University Hospital Southampton NHS Foundation Trust, Southampton, GBR; 3 Internal Medicine, University of Medicine (1), Yangon, Yangon, MMR

**Keywords:** checkpoint inhibitor, haemophagocytic lymphohistiocytosis, hlh, immune-related adverse event, immunotherapy, ipilimumab, mesothelioma, nivolumab

## Abstract

Haemophagocytic lymphohistiocytosis (HLH) is a rare but potentially fatal hyperinflammatory syndrome caused by dysregulated immune activation. With the increasing use of immune checkpoint inhibitors (ICIs), HLH has emerged as an uncommon but serious immune-related adverse event.

An 84-year-old woman with malignant pleural mesothelioma developed fever, rigours, collapse, confusion, worsening cytopenias, cholestatic liver dysfunction, marked hyperferritinaemia, hypertriglyceridaemia, and hypofibrinogenaemia after treatment with ipilimumab and nivolumab. Extensive microbiological investigations were negative. She fulfilled five HLH-2004 diagnostic criteria, supporting a diagnosis of probable ICI-associated HLH. Dexamethasone was started, with rapid clinical and biochemical improvement.

This case highlights the importance of recognising HLH as a rare but life-threatening complication of checkpoint inhibitor therapy and supports early corticosteroid treatment, once infection has been reasonably excluded.

## Introduction

Haemophagocytic lymphohistiocytosis (HLH) is a rare but potentially life-threatening hyperinflammatory syndrome characterised by uncontrolled activation of macrophages and T lymphocytes, cytokine excess, and progressive organ dysfunction. The diagnosis of HLH was based on the HLH-2004 diagnostic criteria, which require either a molecular diagnosis consistent with HLH or fulfilment of at least five of eight clinical and laboratory criteria: fever, splenomegaly, cytopenias affecting at least two cell lines, hypertriglyceridaemia and/or hypofibrinogenaemia, haemophagocytosis in bone marrow, spleen, or lymph nodes, low or absent natural killer (NK)-cell activity, elevated soluble CD25 (soluble IL-2 receptor), and hyperferritinaemia (>500 μg/L) [1].

Immune checkpoint inhibitors (ICIs) have transformed the treatment of malignant pleural mesothelioma, and nivolumab plus ipilimumab is an established first-line treatment for unresectable disease [2]. The combination subsequently received regulatory approval by the U.S. Food and Drug Administration for this indication [3].

Although uncommon, HLH has increasingly been recognised as a rare immune-related adverse event associated with ICI therapy [4,5]. Recent reviews and pharmacovigilance studies have further described its clinical characteristics, diagnosis, and management [6-8]. This report describes probable ICI-associated HLH in a patient with malignant pleural mesothelioma treated with dual checkpoint inhibition.

## Case presentation

An 84-year-old woman with malignant pleural mesothelioma was receiving the combination of ipilimumab and nivolumab. Her past medical history included hypertension, gastro-oesophageal reflux disease, osteoarthritis, diverticular disease, and recurrent pleural effusions.

She presented with a four-day history of fever, rigours, nausea, abdominal discomfort, weakness, and collapse associated with transient loss of consciousness. During admission, she became confused and developed visual hallucinations. Initial investigations demonstrated anaemia, thrombocytopenia, lymphopenia, raised inflammatory markers, and deranged liver function tests.

**Figure 1:**
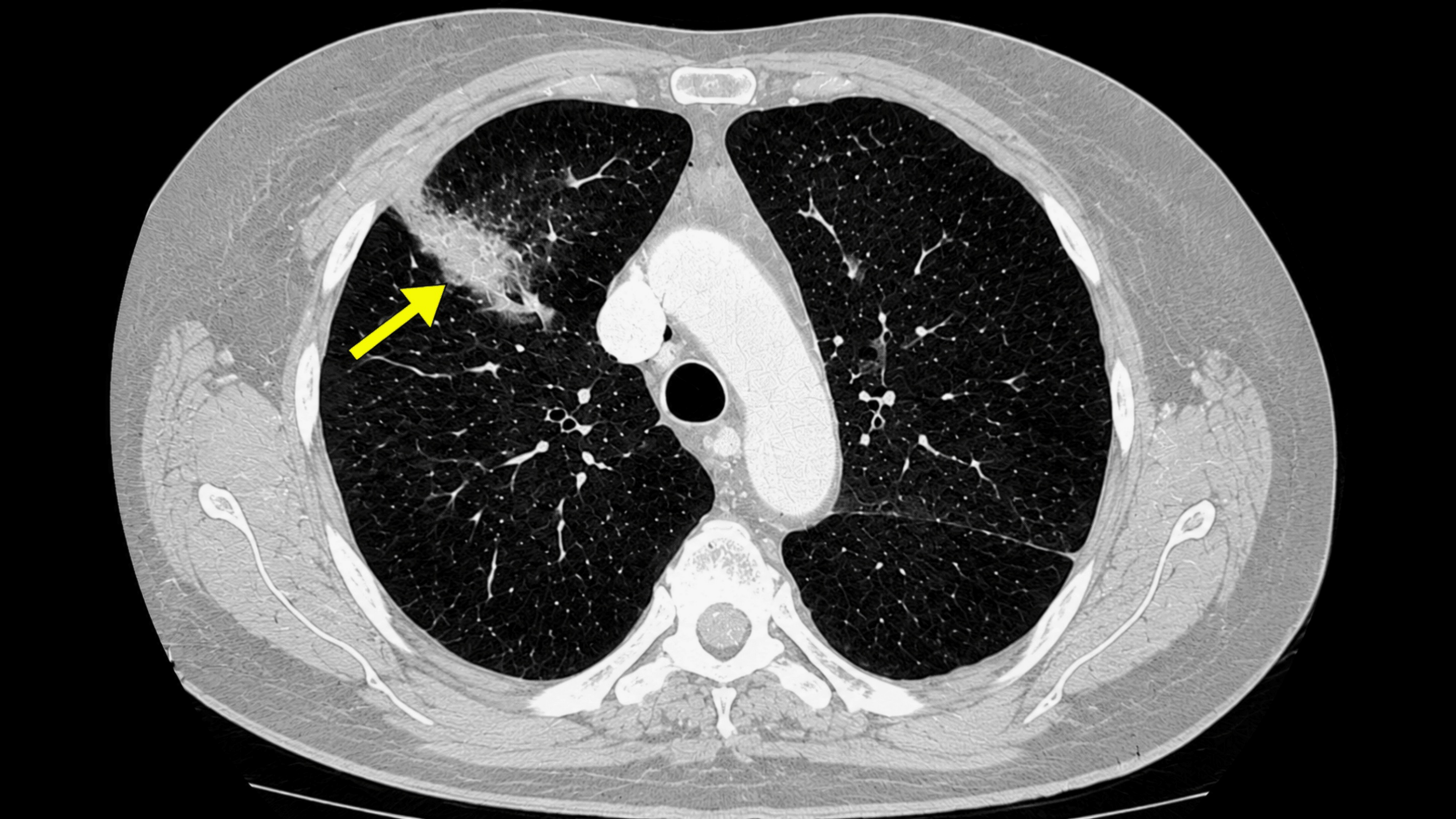
Axial CT pulmonary angiography (lung window) demonstrating a focal peripheral consolidation in the right upper lobe (arrow), which initially raised the differential diagnosis of pneumonia versus pulmonary infarction. CT: computed tomography

**Figure 2:**
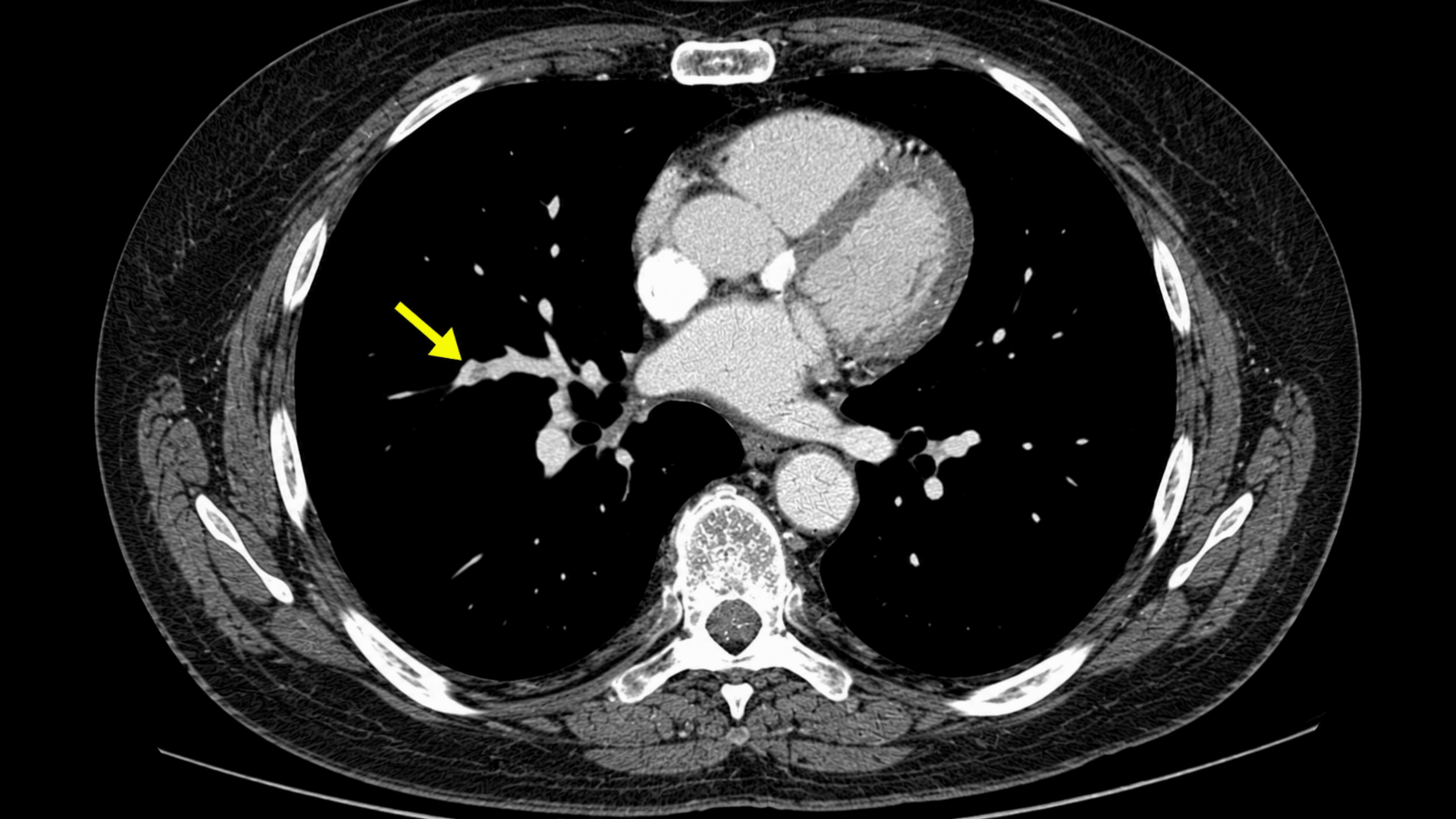
Axial CT pulmonary angiography (mediastinal window) demonstrating a filling defect within a segmental branch of the right upper lobe pulmonary artery (arrow), consistent with acute segmental pulmonary embolism. CT: computed tomography

Computed tomography pulmonary angiography (CTPA) demonstrated a focal peripheral consolidation in the right upper lobe (Figure 1) and a filling defect within a segmental branch of the right upper lobe pulmonary artery, consistent with acute pulmonary embolism (Figure 2). CT of the abdomen and pelvis showed no acute intra-abdominal pathology. She was initially treated for presumed sepsis with broad-spectrum antibiotics, including meropenem, and therapeutic anticoagulation for the acute segmental pulmonary embolism in accordance with local guidelines, with no bleeding complications during admission. Despite antimicrobial therapy, she remained febrile with persistent inflammation, worsening cytopenias, and progressive cholestatic liver dysfunction. Microbiological investigations, including blood cultures, urine cultures, stool bacterial polymerase chain reaction, respiratory viral polymerase chain reaction, and *Clostridioides difficile* testing, were negative.

Further investigations demonstrated marked hyperferritinaemia, hypertriglyceridaemia, hypofibrinogenaemia, elevated lactate dehydrogenase, and progressive pancytopenia, raising suspicion for HLH. Pertinent blood results before initiation of corticosteroid therapy are shown in Table 1.

**Table 1:** Pertinent blood results before dexamethasone therapy.

Parameter	Result	Reference range
Ferritin	>15,000 μg/L	15-150 μg/L
Triglycerides	3.36 mmol/L	<1.7 mmol/L
Fibrinogen	1.2 g/L	2.0-4.0 g/L
Lactate dehydrogenase (LDH)	812 U/L	135-225 U/L
C-reactive protein (CRP)	190 mg/L	<5 mg/L
Haemoglobin (Hb)	88 g/L	115-165 g/L
Platelets	70 × 10⁹/L	150-400 × 10⁹/L
White blood cell (WBC)	3.5 × 10⁹/L	4-11 × 10⁹/L
Neutrophils	2 × 10⁹/L	2-7.5 × 10⁹/L
Lymphocytes	0.8 × 10⁹/L	1-4 × 10⁹/L

The patient fulfilled five of the eight HLH-2004 diagnostic criteria: fever, cytopenias affecting at least two cell lines, hypertriglyceridaemia, hypofibrinogenaemia, and ferritin greater than 500 μg/L. Splenomegaly was absent, haemophagocytosis was not demonstrated, and NK-cell activity and soluble CD25 were not available. In the context of recent dual checkpoint inhibition and negative microbiological testing, probable ICI-associated HLH was diagnosed.

Dexamethasone 10 mg twice daily (10 mg BD) was commenced, resulting in rapid clinical improvement. Fever resolved, mental state normalised, liver biochemistry improved, and haematological parameters gradually recovered. Laboratory trends following steroid therapy are shown in Table 2.

**Table 2:** Serial haematological, liver, and renal biochemical parameters following initiation of dexamethasone therapy. Hb, haemoglobin; WBC, white blood cell count; ALP, alkaline phosphatase; ALT, alanine aminotransferase

Day	Hb (g/L), 115-165	WBC (×10⁹/L), 4.0-11.0	Neutrophils (×10⁹/L), 2.0-7.5	Lymphocytes (×10⁹/L), 1.0-4.0	Platelets (×10⁹/L), 150-400	Albumin (g/L), 35-50	ALP (U/L), 30-130	ALT (U/L), 7-35	Bilirubin (µmol/L), <21	Creatinine (µmol/L), 45-90	eGFR (mL/min/1.73 m²), ≥60	Urea (mmol/L), 2.5-7.8
Day 3	83	5.2	4.2	0.6	420	30	441	62	18	57	82	8.5
Day 4	79	6.5	4.1	1.4	413	25	333	52	17	47	87	6.2
Day 5	87	6.1	3.8	1.3	432	29	376	55	20	47	87	7
Day 7	99	6.3	3.3	2	371	32	333	52	21	55	83	9.3
Day 8	95	5.8	3.5	1.5	346	31	261	47	17	60	80	8.4
Day 10	88	4.5	2.3	1.4	259	32	211	46	16	58	81	8.8

A summary of the HLH-2004 criteria fulfilled in this case is shown in Table 3.

**Table 3:** HLH-2004 diagnostic criteria. HLH: haemophagocytic lymphohistiocytosis

Criterion	Present
Fever >38.5°C	Yes
Cytopenias affecting ≥2 cell lines	Yes
Hypertriglyceridaemia	Yes
Hypofibrinogenaemia	Yes
Ferritin >500 μg/L	Yes
Splenomegaly	No
Haemophagocytosis	Not demonstrated
Natural killer (NK)-cell activity/sCD25	Not available

Following sustained clinical and biochemical improvement, she was discharged home on Day 10 of admission with oncology follow-up and a tapering course of dexamethasone.

## Discussion

HLH is a rare but potentially fatal immune-related adverse event associated with ICI therapy [4-8]. It results from excessive activation of macrophages and cytotoxic T lymphocytes, leading to uncontrolled cytokine release, tissue injury, and multiorgan dysfunction [1]. Because its clinical presentation frequently overlaps with severe infection, disease progression, or other immune-related adverse events, early diagnosis remains challenging.

In our patient, the initial working diagnosis was sepsis because of fever, elevated inflammatory markers, and focal pulmonary consolidation on CT imaging. However, persistent clinical deterioration despite broad-spectrum antibiotics, together with negative microbiological investigations, worsening cytopenias, marked hyperferritinaemia, hypertriglyceridaemia, hypofibrinogenaemia, and progressive liver dysfunction, raised a strong suspicion of HLH. The rapid clinical and biochemical response following initiation of dexamethasone further supported an immune-mediated process rather than uncontrolled infection.

The HLH-2004 criteria remain the most widely used diagnostic framework for HLH, particularly when specialised investigations are unavailable [1]. Although bone marrow examination, soluble CD25 measurement, and NK-cell activity testing may provide additional diagnostic information, treatment should not be delayed when there is a high clinical suspicion of HLH. In our patient, the diagnosis was established based on fulfilment of five of the eight HLH-2004 diagnostic criteria, together with the characteristic clinical and biochemical findings, allowing prompt initiation of corticosteroid therapy. Furthermore, the absence of demonstrable haemophagocytosis does not exclude HLH, as haemophagocytosis may be absent early in the disease course.

Published reports of ICI-associated HLH describe similar clinical presentations characterised by persistent fever, cytopenias, hyperferritinaemia, hypertriglyceridaemia, liver dysfunction, and elevated inflammatory markers following treatment with PD-1, PD-L1, or CTLA-4 inhibitors [4-6,8]. Most reported cases occurred in patients with melanoma, non-small cell lung cancer, renal cell carcinoma, and haematological malignancies receiving nivolumab, pembrolizumab, ipilimumab, or combination immunotherapy. Similar to previously published cases, our patient demonstrated rapid clinical and biochemical improvement following prompt corticosteroid therapy after infection had been reasonably excluded. However, reports of HLH occurring in patients with malignant pleural mesothelioma treated with nivolumab and ipilimumab remain extremely limited, making our case an important addition to the growing literature on this rare but serious immune-related adverse event.

Management of severe immune-related adverse events generally includes withholding ICI therapy and initiating high-dose corticosteroids, with escalation to additional immunosuppressive agents such as anakinra, intravenous immunoglobulin, or etoposide in refractory cases [7]. Consistent with these recommendations, nivolumab and ipilimumab were withheld in our patient, and dexamethasone therapy resulted in rapid resolution of fever, improvement in mental status, recovery of cytopenias, and normalisation of liver biochemistry. She was subsequently discharged with oncology follow-up for consideration of further oncological management. As nivolumab plus ipilimumab continues to be the standard first-line treatment for unresectable malignant pleural mesothelioma [2,3], clinicians should remain vigilant for this rare but potentially life-threatening complication.

## Conclusions

ICI-associated HLH is a rare but potentially life-threatening complication of cancer immunotherapy. Persistent fever, worsening cytopenias, hyperferritinaemia, hypertriglyceridaemia, and hypofibrinogenaemia in a patient receiving checkpoint inhibitors should prompt urgent evaluation for HLH. Early recognition, exclusion of alternative diagnoses such as infection, and prompt initiation of corticosteroid therapy may result in rapid clinical and biochemical improvement. Increased awareness of this rare immune-related adverse event is important as the use of ICIs continues to expand.

## References

[1] Henter JI, Horne A, Aricó M (2007). HLH-2004: diagnostic and therapeutic guidelines for hemophagocytic lymphohistiocytosis. Pediatr Blood Cancer.

[2] Baas P, Scherpereel A, Nowak AK (2021). First-line nivolumab plus ipilimumab in unresectable malignant pleural mesothelioma (CheckMate 743): a multicentre, randomised, open-label, phase 3 trial. Lancet.

[3] (2020). FDA approves nivolumab and ipilimumab for unresectable malignant pleural mesothelioma. https://www.asco.org/practice-policy/policy-issues-statements/asco-in-action/fda-approves-nivolumab-and-ipilimumab.

[4] Sadaat M, Jang S (2018). Hemophagocytic lymphohistiocytosis with immunotherapy: brief review and case report. J Immunother Cancer.

[5] Ali SB, Kuss B, Karapetis C, Hughes T, Smith A (2023). Immune checkpoint inhibitor-associated hemophagocytic lymphohistiocytosis in a patient with chronic lymphocytic leukemia. Immunotherapy.

[6] Xu Z, Li H, Yu X, Luo J, Zhang Z (2024). Clinical characterization of hemophagocytic lymphohistiocytosis caused by immune checkpoint inhibitors: a review of published cases. Hematology.

[7] Schneider BJ, Naidoo J, Santomasso BD (2021). Management of immune-related adverse events in patients treated with immune checkpoint inhibitor therapy: ASCO guideline update. J Clin Oncol.

[8] Diaz L, Jauzelon B, Dillies AC, Le Souder C, Faillie JL, Maria AT, Palassin P (2023). Hemophagocytic lymphohistiocytosis associated with immunological checkpoint inhibitors: a pharmacovigilance study. J Clin Med.

